# Supplementary material for: Convis: A Toolbox to Fit and Simulate Filter-Based Models of Early Visual Processing
Source: Front Neuroinform. 2018 Mar 7;12:9. doi: 10.3389/fninf.2018.00009 (PMC5845886; doi:10.3389/fninf.2018.00009)
Supplement: Supplementary file 1 [file Presentation1.pdf]

Convis: A Toolbox To Fit and Simulate Filter-based Models of Early Visual Processing

Supplementary Material

Jacob Huth, Timothée Masquelier, Angelo Arleo

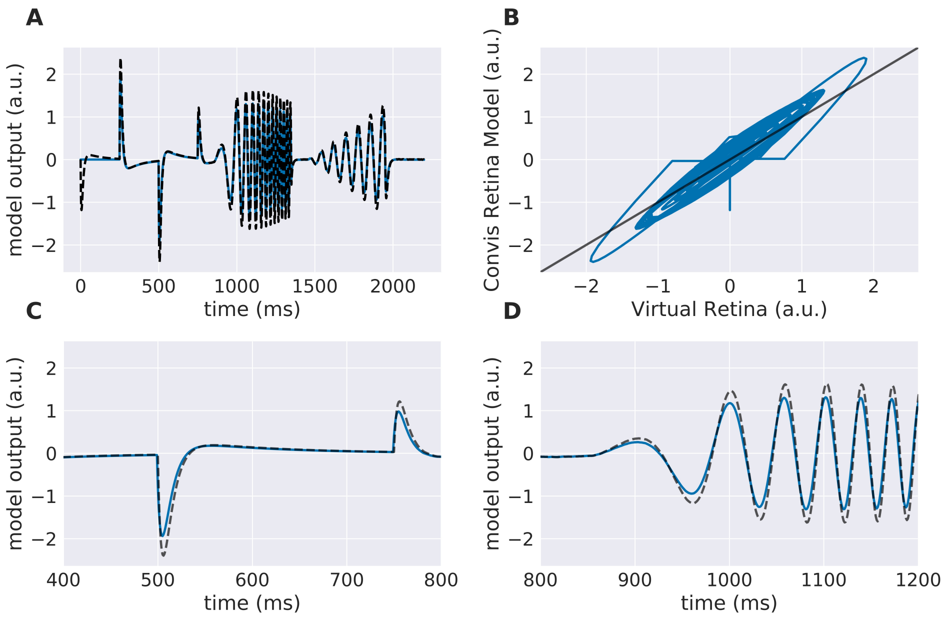

Figure 11: Comparison of Virtual Retina OPL stage (dashed line) and the OPL stage of the Convis Retina model (solid line): The stimulus is a "chirp" (see Figure 1). (A) shows the complete trial, (C) and (D) show details; (B) compares the trace of the original VirtualRetina to the new model. Due to numerical differences between the method of recursive filtering and convolutional filtering, the full-convolution OPL stage differs slightly from the original trace for very abrupt changes.

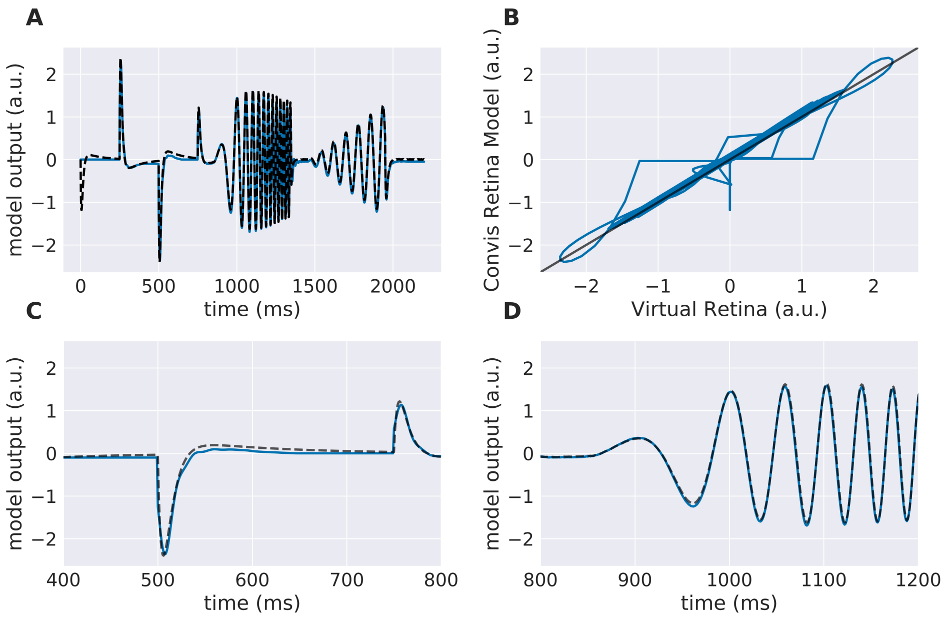

Figure 12: Comparison of Virtual Retina OPL stage (dashed line) and a linear model with a single convolutional filter (solid line): The OPL stage fitted to the desired response instead of using configuration values can reproduce the response of the VirtualRetina OPL faithfully. (A) shows the complete trial, (C) and (D) show details; (B) compares the trace of the original VirtualRetina to the new model.

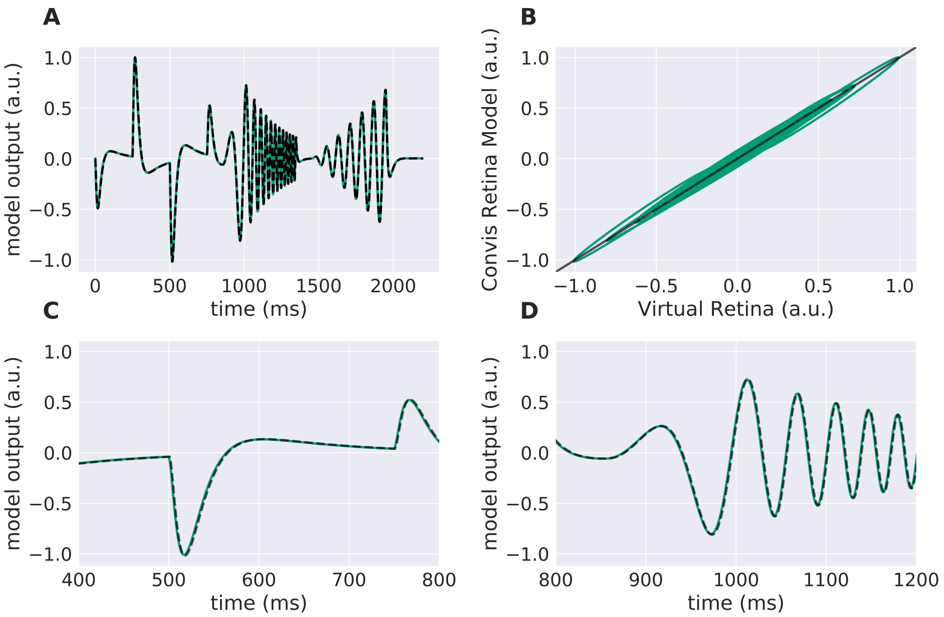

Figure 13: Comparison of Virtual Retina Bipolar stage (dashed line) and the Bipolar stage of the Convis Retina model (solid line). The stimulus is a “chirp” (see Figure 1). (A) shows the complete trial, (C) and (D) show details; (B) compares the trace of the original VirtualRetina to the new model.

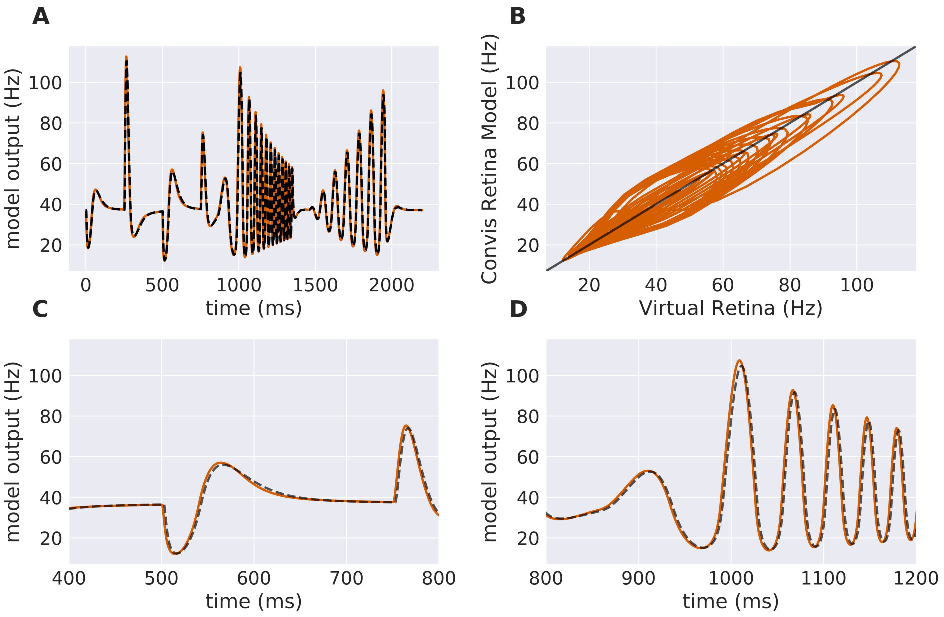

Figure 14: Comparison of Virtual Retina Ganglion Layer stage (dashed line) and the Ganglion Layer stage of the Convis Retina model (solid line). The stimulus is a “chirp” (see Figure 1). (A) shows the complete trial, (C) and (D) show details; (B) compares the trace of the original VirtualRetina to the new model.

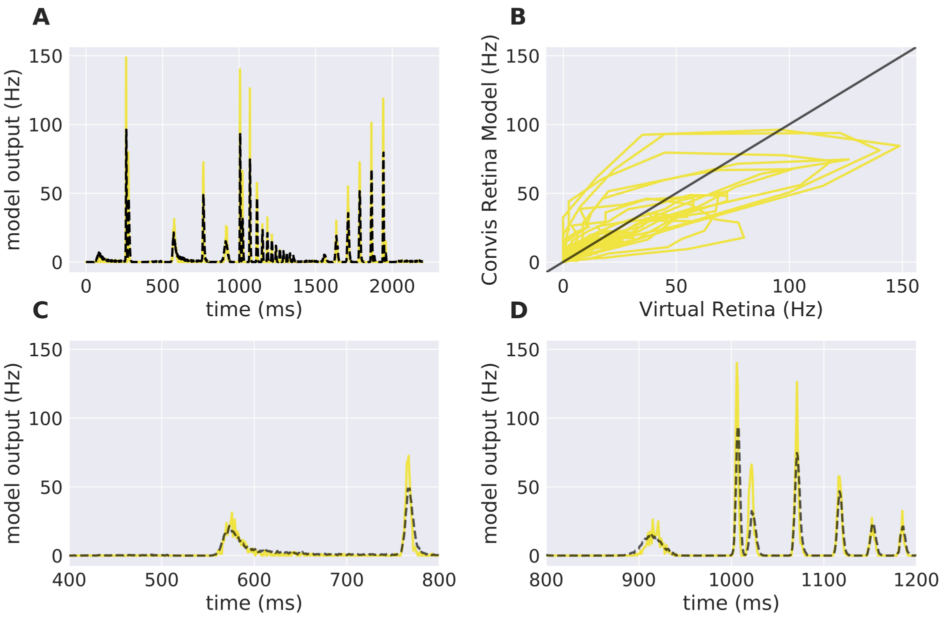

Figure 15: Comparison of Virtual Retina Ganglion On Spiking Layer stage (dashed line) and the GanglionSpiking stage of the Convis Retina model (solid line). The stimulus is a “chirp” (see Figure 1). (A) shows the complete trial, (C) and (D) show details; (B) compares the trace of the original VirtualRetina to the new model.

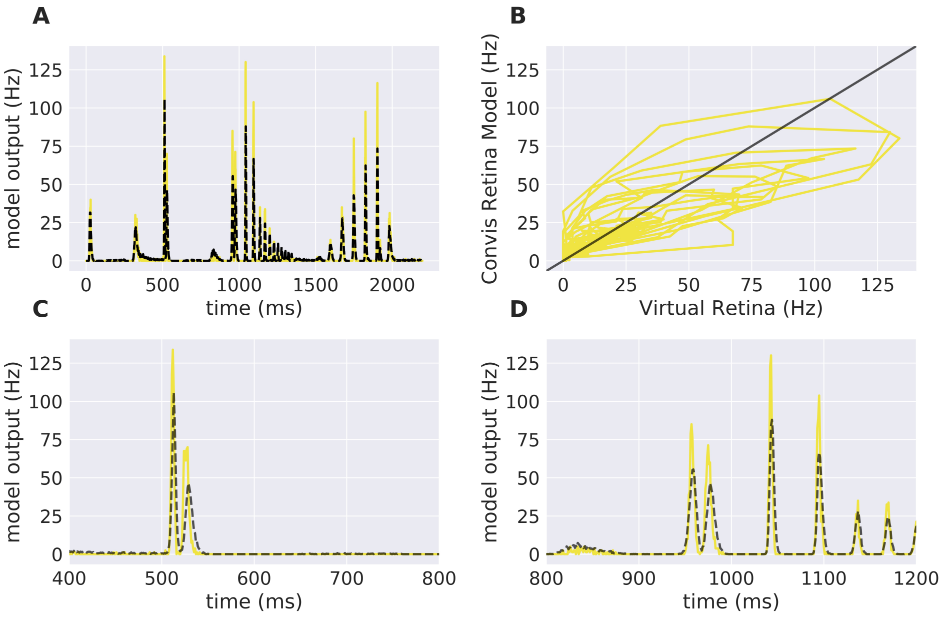

Figure 16: Comparison of Virtual Retina Ganglion Off Spiking Layer stage (dashed line) and the GanglionSpiking stage of the Convis Retina model (solid line). The stimulus is a “chirp” (see Figure 1). (A) shows the complete trial, (C) and (D) show details; (B) compares the trace of the original VirtualRetina to the new model.

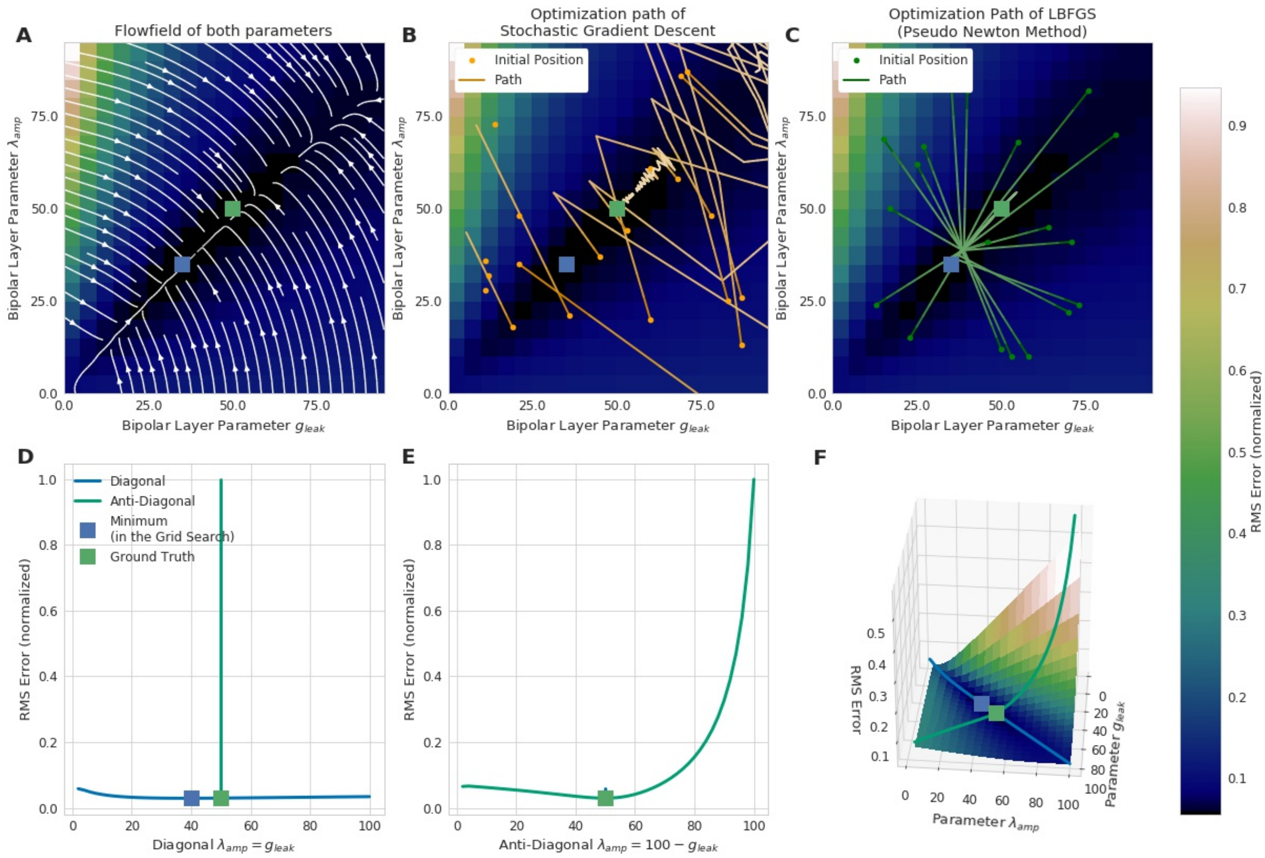

Figure 17: The interaction of two parameters in the Convis Retina model poses a problem for naive optimization methods. We visualized a grid search for the true parameters of a ground-truth model in terms of the gradients with respect to each of the parameters and the error function with an overlay of the flow field resulting from the two gradient functions. The blue square shows the global minimum of the grid search (which does not correspond to the ground truth values of the two parameters since the parameters lie between the grid as can be expected when parameters are unknown). (A) shows the combination of both gradients. Most gradients do not point directly at the true minimum or at the ground truth, but rather at the a "valley" along the diagonal. (B) and (C) show the path of two optimizer algorithms when searching for the parameters. The stochastic gradient descent method has trouble finding the minimum since the direction of the gradients carry only little information. The optimizer "bounces" between the walls and started to escape to increasingly large values (trials were stopped once the parameters left a certain region). The pseudo Newton method of the LBFGS shows a quick convergence towards one point in the valley and a subsequent approach to the true values. So it can be noted that although the error plane is convex, grid search found a minimum, which was not very close to the true minimum in the parameter space, gradient descent had great difficulties finding even the valley. Gradient descent methods need very sophisticated learning rate schedulers to adapt to the large range of gradients. LBFGS converged to the same point independent of initial condition, which is due to the self guided exploration the methods uses to approximate the error surface. But to get closer to the true parameters, more iterations were needed. (D) and (E) show the profile of the diagonal and anti-diagonal (as shown in (F)). The error along the diagonal is much shallower than the gradients on both sides of the valley. (F) shows the error in a surface plot with the diagonal and anti-diagonal highlighted.
